# A Phylogenomic Study of *Acanthamoeba polyphaga* Draft Genome Sequences Suggests Genetic Exchanges With Giant Viruses

**DOI:** 10.3389/fmicb.2018.02098

**Published:** 2018-09-06

**Authors:** Nisrine Chelkha, Anthony Levasseur, Pierre Pontarotti, Didier Raoult, Bernard La Scola, Philippe Colson

**Affiliations:** Institut de Recherche pour le Développement, Assistance Publique – Hôpitaux de Marseille, Microbes, Evolution, Phylogeny and Infection, and Institut Hospitalo-Universitaire – Méditerranée Infection, Aix-Marseille Université, Marseille, France

**Keywords:** *Acanthamoeba polyphaga*, *Acanthamoeba*, giant viruses, mimivirus, draft genome sequences, horizontal gene transfer, nucleotide sequence transfer

## Abstract

*Acanthamoeba* are ubiquitous phagocytes predominant in soil and water which can ingest many microbes. Giant viruses of amoebae are listed among the *Acanthamoeba*-resisting microorganisms. Their sympatric lifestyle within amoebae is suspected to promote lateral nucleotide sequence transfers. Some *Acanthamoeba* species have shown differences in their susceptibility to giant viruses. Until recently, only the genome of a single *Acanthamoeba castellanii* Neff was available. We analyzed the draft genome sequences of *Acanthamoeba polyphaga* through several approaches, including comparative genomics, phylogeny, and sequence networks, with the aim of detecting putative nucleotide sequence exchanges with giant viruses. We identified a putative sequence trafficking between this *Acanthamoeba* species and giant viruses, with 366 genes best matching with viral genes. Among viruses, Pandoraviruses provided the greatest number of best hits with 117 (32%) for *A. polyphaga*. Then, genes from mimiviruses, Mollivirus sibericum, marseilleviruses, and Pithovirus sibericum were best hits in 67 (18%), 35 (9%), 24 (7%), and 2 (0.5%) cases, respectively. Phylogenetic reconstructions showed in a few cases that the most parsimonious evolutionary scenarios were a transfer of gene sequences from giant viruses to *A. polyphaga.* Nevertheless, in most cases, phylogenies were inconclusive regarding the sense of the sequence flow. The number and nature of putative nucleotide sequence transfers between *A. polyphaga*, and *A. castellanii* ATCC 50370 on the one hand, and pandoraviruses, mimiviruses and marseilleviruses on the other hand were analyzed. The results showed a lower number of differences within the same giant viral family compared to between different giant virus families. The evolution of 10 scaffolds that were identified among the 14 *Acanthamoeba* sp. draft genome sequences and that harbored ≥ 3 genes best matching with viruses showed a conservation of these scaffolds and their 46 viral genes in *A. polyphaga*, *A. castellanii* ATCC 50370 and *A. pearcei*. In contrast, the number of conserved genes decreased for other *Acanthamoeba* species, and none of these 46 genes were present in three of them. Overall, this work opens up several potential avenues for future studies on the interactions between *Acanthamoeba* species and giant viruses.

## Introduction

*Acanthamoeba* spp. (*Eukaryota*, *Amoebozoa*, *Acanthamoebidae*) are among the most predominant protozoa in soil and water (Rodríguez-Zaragoza, 1994). These amoebae are found in natural or artificial habitats, mostly humid ones, such as the marine environment, sediments, salt lakes, cooling towers, stagnant water, treatment plant sewage, drinking water, or soil (Rodríguez-Zaragoza, 1994). Their ubiquity in water and soil promotes contacts with animals including humans (Sherr and Sherr, 2002; Marciano-cabral and Cabral, 2003). Moreover, *Acanthamoeba* spp. are phagocytic protists that can ingest all particles with a size > 0.5 μm, which includes notably bacteria and fungi (Raoult and Boyer, 2010). Whereas most of these microorganisms are degraded post-internalization, some are able to survive and multiply (Raoult and Boyer, 2010). They are known as amoeba-resisting microorganisms (ARMs) (Greub and Raoult, 2004). Examples of ARMs include human pathogens such as *Legionella pneumophila* or *Mycobacterium* sp., which also resist degradation by macrophages (Greub and Raoult, 2004; Salah et al., 2009).

Due to their giant size, giant viruses of amoebae are ARMs that multiply inside *Acanthamoeba polyphaga* and *A. castellanii* (Colson et al., 2017b). These amoebae have been used in laboratory settings for giant virus isolation during the last 14 years since the discovery of their first representative, Mimivirus (La Scola et al., 2003; Raoult et al., 2004; Colson et al., 2017a). Mimivirus led to the creation of the family *Mimiviridae* and to the discovery of many other giant viruses of amoebae, as well as virophages that replicate in mimivirus factories, and transpovirons integrated in mimivirus genomes (Colson et al., 2017a). Until now, three new families of amoeba-infecting viruses have been described, which include *Mimiviridae*, *Marseilleviridae*, and *Lavidaviridae*, as well as seven new putative lineages consisting in pandoraviruses, pithoviruses, faustoviruses, Mollivirus sibericum, Kaumoebavirus, cedratviruses and Pacmanvirus (Colson et al., 2017b). These giant viruses are commonly found in environmental water and soil and have a broad geographical distribution. They differ from classical viruses and have a complexity similar to that of other microbes. Emblematically, the virions are visible with an optical microscope and can reach 1.5 μm in size, and their genomes harbor between 444 (for a marseillevirus) and 2,544 (for a pandoravirus) genes (Raoult et al., 2004; Colson et al., 2017a).

The hosting by *Acanthamoeba* spp. of several ARMs living sympatrically confers to these microorganisms increased opportunities to exchange sequences between each other and with the amoebal host (Greub and Raoult, 2004; Raoult and Boyer, 2010; Bertelli and Greub, 2012). This is suspected to promote the broad mosaicism and large size of the genomes of giant viruses of amoebae that are in close vicinity with other viruses and microorganisms inside *Acanthamoeba*. It has also been observed that bacteria and viruses living in sympatry in *Acanthamoeba* harbored larger genomes than their closest relatives with an allopatric lifestyle (Raoult and Boyer, 2010). Such sequence exchanges may allow accumulating a substantial gene armory to multiply and compete with other amoeba-resisting microorganisms (Boyer et al., 2011). Consistently, culturing Mimivirus in allopatric conditions on ARM-free *Acanthamoeba* led to a 16% reduction of the viral genome after 100 passages (Boyer et al., 2011).

According to recent findings, the species *A. castellanii* and *A. polyphaga*, which were those used to isolate giant viruses of amoebae, have different levels of tolerance to viruses from different or even similar lineages (Dornas et al., 2015). We observed that some virus lineages relied on specific *Acanthamoeba* species for their replication. For example, pandoraviruses and pithoviruses were only isolated on *A. castellanii* and faustoviruses were only isolated on *Vermamoeba vermiformis* (Khalil et al., 2016; Reteno et al., 2015). Moreover, different mimivirus isolates were obtained from the same sample with different *Acanthamoeba* species (Dornas et al., 2015). These data raise some questions about the relationship between *Acanthamoeba* species and giant viruses of amoebae.

Giant viruses of amoebae have raised a radically new issue regarding their genomic content. Since the description of the Mimivirus, the question of the origin of their genes has arisen. From the onset, genes corresponding to nucleotide sequences putatively transferred from amoebae to viruses were identified, and it was proposed that giant viruses were essentially bags of exogenous genes (Filée et al., 2007; Moreira and Brochier-Armanet, 2008). This assertion is only partially true, since several genes were suspected to be shared by several giant viruses, and the proportion of ORFan genes, for which no source is identified, remains very high in giant viruses (Colson et al., 2017b). Nucleotide sequences from bacterial viruses (bacteriophages), viruses of viruses (virophages), archaeal viruses, or eukaryotic viruses can possibly be transferred inside their host genomes. Regarding amoebae, at present, nucleotide sequence transfers are mostly studied by comparing their genomes to those of giant viruses. *A. castellanii* was the only *Acanthamoeba* species for which we had draft genome sequences (from 2010 to 2015), which was completed using transcriptomic data (Clarke et al., 2013). The study of this genome led to infer sequence exchanges with other eukaryotes and with archaea, bacteria, and viruses of the proposed order Megavirales, which comprises the formerly described Nucleocytoplasmic large DNA viruses (NCLDVs) and giant viruses of amoebae (Yutin et al., 2009; Colson et al., 2013). In this work, we sought to detect and characterize putative nucleotide sequence transfers involving giant virus genomes and the draft genome sequences of a second *Acanthamoeba* species, *A. polyphaga*, which has been one of the most used to isolate giant viruses of amoebae.

## Materials and Methods

### Gene Content of Giant Viruses

The study of the gene trafficking between *A. polyphaga* and giant viruses was carried out by using genes of all annotated genomes described for these viruses at the time of our analysis (including mimiviriruses, marseilleviruses, pandoraviruses, Pithovirus sibericum, Mollivirus sibericum, faustoviruses, phycodnaviruses, ascoviruses, iridoviruses, asfarviruses and poxviruses) (**Supplementary Table S1**).

### Draft Genome Sequences of *Acanthamoeba polyphaga* and Other *Acanthamoeba* Species

The draft genome of *A. polyphaga* ATCC 30872 is publicly available on the NCBI website^1^ (accession: PRJEB7687). It is part of the project «Phylogenomics of *Acanthamoeba* species» (Institute of Integrative Biology, University of Liverpool), along with the draft genomes of 13 other *Acanthamoeba* species. The *A. polyphaga* ATCC 30872 draft genome contains 224,482 scaffolds with a total length of 120.6 megabases (Mb). The 14,974 genes identified in the genome of *A. castellanii* Neff with the support of transcriptomic data (Clarke et al., 2013) were used for comparative genomic analyses.

### Optimization of Assembly of the *Acanthamoeba* Draft Genome Sequences

We used the CLC Genomics Workbench software (version 7.5)^2^ to reassemble the draft genome sequences of *A. polyphaga* and some other *Acanthamoeba* species, including another *A. castellanii* strain (ATCC 50370). The default kmer size of 50 was used.

### Gene Prediction, Functional Annotation, and Analysis of Taxonomical Distribution

Gene prediction for the draft genome sequences of *A. polyphaga* and other *Acanthamoeba* species was performed using the Prodigal program, as we searched for sequences best matching those of giant viruses. This tool identifies ribosome binding sites to localize translation initiation positions and localizes precisely the 3′ end of each gene (Hyatt et al., 2010). The hits identified as those best matching with giant virus genes were then checked and compared with the 47,246 genes predicted for *A. polyphaga* using GeneMarkES, a program developed specifically for eukaryotes (Lomsadze et al., 2005). For the functional annotation, sequences homologous with ORFs predicted from non-redundant scaffolds were searched for in the NCBI GenBank protein sequence database (nr) using the BLASTp program (Altschul et al., 1990). In addition, ORFs were identified through BLASTp searches (with an *e*-value threshold of 0.1) in the database of Clusters of Orthologous Groups of proteins (COGs) of the NCBI (Tatusov, 2000). Taxonomical origins were determined using MEGAN6 (Huson et al., 2016).

### Comparative Genomic Analyses

Predicted protein sequences of *A. polyphaga* were compared with those from giant viruses and those predicted from the draft genome sequences of the 13 other species of *Acanthamoeba* (**Supplementary Table S2**). The orthologous genes (≥100 amino acids) of *A. polyphaga* and the other *Acanthamoeba* were identified using the Proteinortho program (Lechner et al., 2011). Phylogenetic reconstructions were then performed using two sets of aligned sequences. First, 16 draft genome sequences of *Acanthamoeba* strains classified in 14 species including two *A. polyphaga* strains and two *A. castellanii* strains were aligned by using the progressive Mauve program (Darling et al., 2010). Second, the 18S ribosomal DNA sequences from 33 different *Acanthamoeba* strains were retrieved, including those of the 16 *Acanthamoeba* strains classified in 14 *Acanthamoeba* species. The 18S ribosomal DNA sequences were obtained by sequencing the complete 18S ribosomal DNA from strains available in our laboratory, and for non-available strains, the sequences were retrieved from the NCBI GenBank database or directly from the *Acanthamoeba* draft genome sequences. Nucleotide sequences alignments were performed with the MUSCLE program (Edgar, 2004). Phylogenetic trees were constructed using FastTree (Price et al., 2010).

We investigated more specifically any possible occurrences of horizontal gene transfers (HGT), i.e., the gene trafficking between this amoeba and giant viruses. *A. polyphaga* proteins which had as significant hit a giant virus sequence were used as queries to search into the NCBI GenBank non-redundant protein sequence database (nr). Phylogenetic analyses were performed to confirm suspicion of HGT for the genes showing the highest level of sequence similarity with a viral homolog. Amino acid sequences alignments were performed with the MUSCLE program. Phylogenetic trees were constructed using FastTree. Ancestral major capsid protein (MCP) sequences were predicted using the MEGA6 program^3^. Additionally, similarity searches were performed by using the tBLASTn program for all ORFs of giant viruses, virophages and transpovirons against the draft genome sequences of *A. polyphaga*. The following criteria were used: a percentage of amino acid identity ≥ 30%; an *e*-value ≤ 1e-2; and a percentage of coverage of aligned sequences ≥ 30%. Finally, results from similarity searches were formatted to create networks of gene trafficking using the Cytoscape tool (Smoot et al., 2011). This software was also used to generate a network between protein sequences from two giant viruses of amoebae, Pandoravirus dulcis and Pandoravirus salinus, and from the draft genome sequences of *A. polyphaga* and *A. castellanii* ATCC 50370. Finally, a ‘rhizome’ of genes was determined for a few *A. polyphaga* genes whose best hit was a giant virus. This information was obtained by performing BLASTp searches with fragments of this gene obtained by fenestrating its amino acid sequence with a window of 40 amino acids and a sliding step of 20 amino acids. The representation of the mosaicism of these genes was built using the Circos tool^4^.

We identified 115 ORFs (i) best matching with a giant virus gene, (ii) larger than 100 amino acids, and (iii) present in scaffolds that harbor a majority of ‘non-viral’ ORFs. For these 115 ORFs, we performed BLASTp searches against the GenBank protein sequence database nr and a tBLASTn search against all 14 *Acanthamoeba* draft genome sequences. Then, we merged the results of these two BLAST searches by creating a database with significant sequence hits (*e*-value < 1e-4, length of query and subject sequence alignments > 100 amino acids), and performed another BLASTp search for the 115 ORFs against this database. We thereafter examined if best hits were exclusively or in majority sequences from giant viruses or *Acanthamoeba* spp. or both and performed a phylogenetic reconstruction to determine the sense of the nucleotide sequence transfers between these organisms.

### Synteny Analysis in the Draft Genome Sequences of the 14 *Acanthamoeba* Species of *A. polyphaga* Genes for Which the Best Match Is a Giant Virus Gene

Synteny preservation of genes from *A. polyphaga* for which the best match is a giant virus gene was evaluated in the draft genome sequences available for the 13 other *Acanthamoeba* species. For this purpose, we selected 10 *A. polyphaga* scaffolds that carried at least 3 such genes, then searched for scaffolds harboring similar genomic sequences in other *Acanthamoeba* species and strains. Finally, we determined whether *A. polyphaga* genes for which the best match is a giant virus gene were present in the genome scaffolds from other species and strains, and whether these genes were in synteny in the different *Acanthamoeba* draft genome sequences.

## Results

### Improvement of the Assembly of the *Acanthamoeba* Draft Genome Sequences

The estimated size of the *A. polyphaga* draft genome was 120.6 Mb, compared to 115.3 Mb for the draft genome sequences of *A. castellanii* ATCC 50370, and 41 Mb for the *A. castellanii* Neff genome (Clarke et al., 2013). Using CLC Genomics Workbench, the number of contigs for the *A. polyphaga* draft genome sequences was reduced from 224,482 to 56,709. Contig number reduction was in the same order of magnitude for *A. castellanii* ATCC 50370 (from 221,748 to 56,469) (**Table 1**). We obtained a statistically significant reduction in the average number (±standard deviation) of contigs for the 14 draft genome sequences of *Acanthamoeba* (*p* < 1e-3, ANOVA test) (**Supplementary Figure S1**).

**Table 1:** Assembly statistics for the draft genome sequences of the two species *A. polyphaga* and *A. castellanii* using the CLC software.

	*A. polyphaga*		*A. castellanii*	
	Initial draft genome	New assembly	Initial draft genome	New assembly
Genome size (Mb)	120.6	123.8	115.3	121.2
Number of contigs	224,482	56,709	221,748	56,469
Mean length (nucleotides)	519	2147	536	2184
N50	1,362	3,982	1,454	4,103
Proportion of contigs < 1,000 bp (%)	86.9	44.1	86.8	44.2
Proportion of contigs > 10,000 bp (%)	0.19	2.56	0.26	2.80
Proportion of contigs < 100,000 bp (%)	0.0009	0	0.0004	0.0018

*bp, base pairs; N50, 50% of the genome is in contigs larger than this size.*

### *Acanthamoeba polyphaga* Gene Content and Comparison With the Gene Content of Other *Acanthamoeba* Species

Gene prediction performed for the *A. polyphaga* draft genome sequences detected a substantial number of ORFs (equal to 310,496) shorter than 50 amino acids (but greater than 35 amino acids). The number of predicted ORFs with a size comprised between 50 and 100 amino acids or larger than 100 amino acids was also considerable, being of 223,728 and 97,092, respectively (**Supplementary Table S2**). Comparison with the *A. castellanii* Neff gene set showed that 97.2% of its genes were detected in the *A. polyphaga* draft genome sequences. The same proportion (97.2%) of *A. castellanii* Neff genes was detected in the draft genome sequences of *A. castellanii* ATCC 50370. These results indicate that a large majority of *A. castellanii* Neff transcribed genes are present in these *Acanthamoeba* sp. strains (**Supplementary Table S3**). The 421 *A. castellanii* Neff genes that were not detected in the *A. polyphaga* draft genome sequences included mostly genes encoding hypothetical proteins [383 genes (91%)]. Other genes included proteins encoding the six MCP described in the *A. castellanii* Neff genome (Maumus and Blanc, 2016), three NAD-dependent epimerase/dehydratase family proteins, two HEAT repeat domains containing proteins, two Rho termination factor domain containing proteins, a chitin synthase, and a putative autophagy protein (**Supplementary Table S4**). In addition, 48,583 (78.6%) of the 61,786 orthologous groups of genes found in the *A. polyphaga* draft genome sequences were also detected in the *A. castellanii* ATCC 50370 draft genome sequences (**Supplementary Figure S2**). The number of non-ORFan genes and ORFan genes larger than 100 amino acids in the *A. polyphaga* draft genome sequences was equal to 58,185 (70.4%) and 24,484 (29.6%), respectively (**Table 2**). Similar proportions were found for the draft genome sequences of *A. castellanii* ATCC 50370, with 56,920 ORFan genes (69.2%) and 25,390 non-ORFan genes (30.8%).

**Table 2:** Gene annotation for species *A. polyphaga* and *A. castellanii*.

Scaffolds/ORFs	*Acanthamoeba polyphaga*	*Acanthamoeba castellanii*
Scaffolds (draft genome)	224,482	221,748
ORFs annotated (> 100 aa)	58,185	56,920
ORFans (> 100 aa)	24,484	25,390

*aa, amino acids. The number of annotated ORFs as well as ORFans, considering only the sequences larger than 100 amino acids.*

Phylogenetic reconstruction based on 18S ribosomal genes (**Supplementary Figure S3**) and the tree based on similarities between the 14 draft genome sequences and the genome of *A. castellanii* Neff (**Supplementary Figure S4**) showed that the *A. castellanii* ATCC 50370 and Neff are not clustered together. In contrast, the *A. castellanii* ATCC 50370 is clustered with *A. polyphaga* and *A. pearci*, *A. pearci* being the isolate closest to *A. polyphaga*. These findings suggest that the genomes of *A. castellanii* Neff and those of *A. castellanii* ATCC 50370 belong to different species. We further checked for similarities between 18S ribosomal DNA sequences from the draft genome sequences of *A. polyphaga* analyzed here and the sequence AY026244 from *A. polyphaga* ATCC 30872. We observed that 18S ribosomal DNA sequences from both strains were not clustered together, which questions the accuracy of the identification of one or both genomes.

### Taxonomical Distribution of *Acanthamoeba polyphaga* Genes and Possible Gene Trafficking Between *Acanthamoeba* spp. and Giant Viruses

**FIGURE 1:**
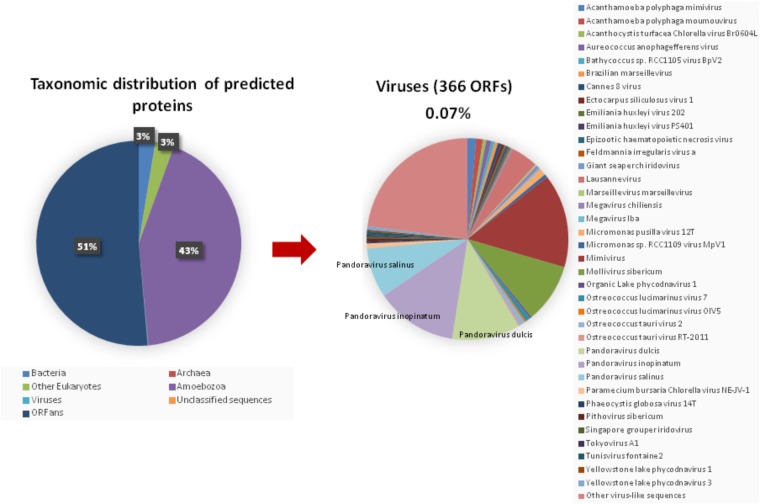
Taxonomic distribution of the predicted proteins in *A. polyphaga*. **(Left)** All predicted proedicted proteins; **(right)** viral proteins. Other virus-like sequences than giant viruses are represented, encompassing the viral families or groups: *Herpesviridae*, *Baculoviridae*, *Podoviridae*, *Nimaviridae, haloviruses*, *Dicistroviridae*, *Paramyxoviridae*, *Totiviridae*, and *Retroviridae*, and Haloviruses.

**FIGURE 2:**
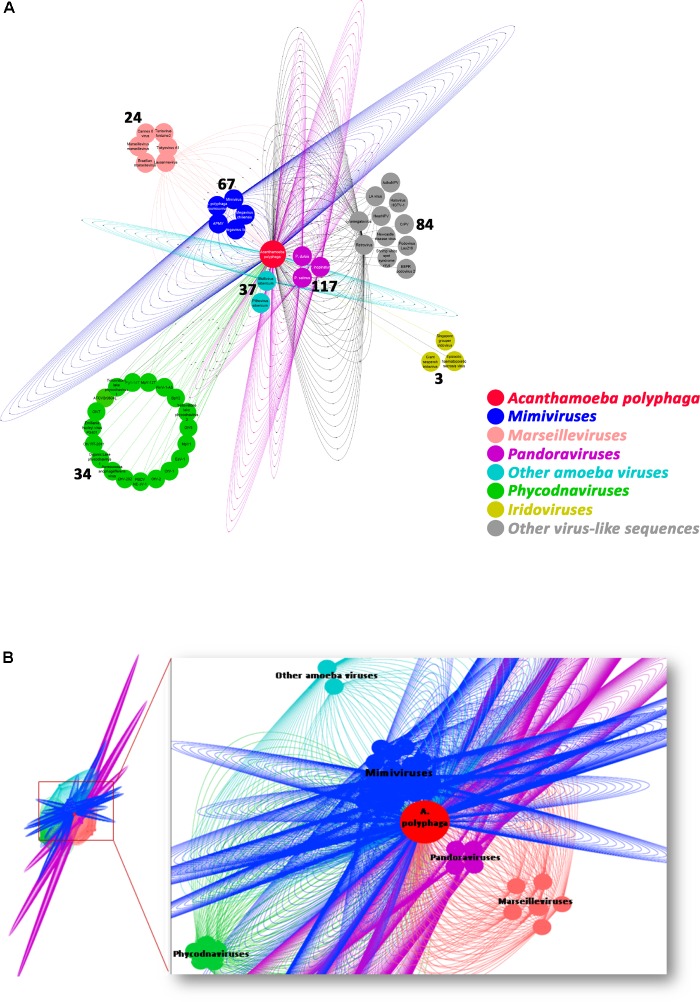
Network of viral genes in *A. polyphaga* **(A)** and gene trafficking between *A. polyphaga* and giant viruses **(B)**. **(A)** genes were classified regarding their viral families, and the number of exchanged genes was indicated for each group of viruses; **(B)** the 1,797 genes were classified with respect to their viral families, and represented by a different color: pandoraviruses in pink; mimiviruses in dark blue; marseilleviruses in light orange; light blue includes other amoeba viruses (Mollivirus sibericum and Pithovirus sibericum); and phycodnaviruses in green.

The taxonomical distribution of the best BLAST hits obtained for the *A. polyphaga* proteins indicated that 43% belong to *Amoebozoa* (98% of them belonging to *A. castellanii* Neff), 3% belong to eukaryotes other than *Amoebozoa*, 3% belong to bacteria, 3% to archaea, and 51% were identified to be ORFans. A total of 366 genes (0.07%) had viral genes as best match, which suggests an important gene trafficking between amoebae and their infecting viruses (**Figures 1**, **2A**). A total of 41 (11%) of the 366 viral genes in *A. polyphaga* were found to match with genes transcribed in *A. castellanii* Neff. The functions of these 366 *A. polyphaga* genes are mostly related to replication, recombination and repair [COG category L (18%)]; then signal transduction [T (16%)]; general function [R (15%)]; post-translational modification [O (12%)]; transcription [K (9%)]; and molecule transport and metabolism [E, F, G, H and P (17%)] (**Table 3**). Most of these 366 genes belong to giant viruses of the proposed order Megavirales. Pandoraviruses were the giant viruses that provided the greatest number of best hits with 117 (32%) for *A. polyphaga*. Then, genes from mimiviruses, Mollivirus sibericum, marseilleviruses, and Pithovirus sibericum, were best hits in 67 (18%), 37 (10%), 24 (7%), and 2 (0.5%) cases, respectively. Among other viral genes, there were 34 genes from phycodnaviruses. Among other viral genes, there were 29 genes from phycodnaviruses. A similar number of viral genes (356) was identified in the draft genome sequences of *A. polyphaga* than in those of *A. castellanii* ATCC 50370 (**Supplementary Table S5**). The 47,246 genes predicted for *A. polyphaga* by using the GeneMarkES program were compared to the 366 genes best matching with virus genes, and this comparison showed that coverage inferior to 50% was only observed for 25 genes (6.8%), while 67 genes did not match with any of the available 47,246 genes. A same analysis was specifically carried out using only available ORFomes from all giant viruses isolated on *Acanthamoeba* spp., allowing the identification of 1,797 genes in the *A. polyphaga* draft genome sequences (1.9% of the 97,092 ORFs larger than 100 amino acids) with a giant viral homolog. In accordance with our previous findings, pandoravirus genes were the most abundant of these giant viral homologous genes, before mimiviruses, marseilleviruses, other giant viruses of amoebae, and phycodnaviruses. A substantial level of gene trafficking between *A. polyphaga* and giant viruses was illustrated by a sequence network that made it possible to observe the number and the nature of genes involved in putative nucleotide sequence transfers (**Figure 2B**).

**Table 3:** Distribution of the genes predicted from the draft genome sequences of *A. polyphaga* with a viral gene as best hit in the functional “COG” categories.

COG functional category	Function	Number of genes
A	RNA processing and modification	1
E	Amino acid transport and metabolism	4
F	Nucleotide transport and metabolism	3
G	Carbohydrate transport and metabolism	1
H	Coenzyme transport and metabolism	3
J	Translation, ribosomal structure and biogenesis	2
K	Transcription	9
L	Replication, recombination and repair	19
M	Cell wall/membrane/envelope biogenesis	1
O	Post-translational modification, protein turnover, chaperones	13
P	Inorganic ion transport and metabolism	7
R	General function prediction only	16
S	Function unknown	5
T	Signal transduction	17
U	Intracellular trafficking, secretion, and vesicular transport	2
V	Defense mechanisms	1

*COG, cluster of orthologous groups of proteins.*

**FIGURE 3:**
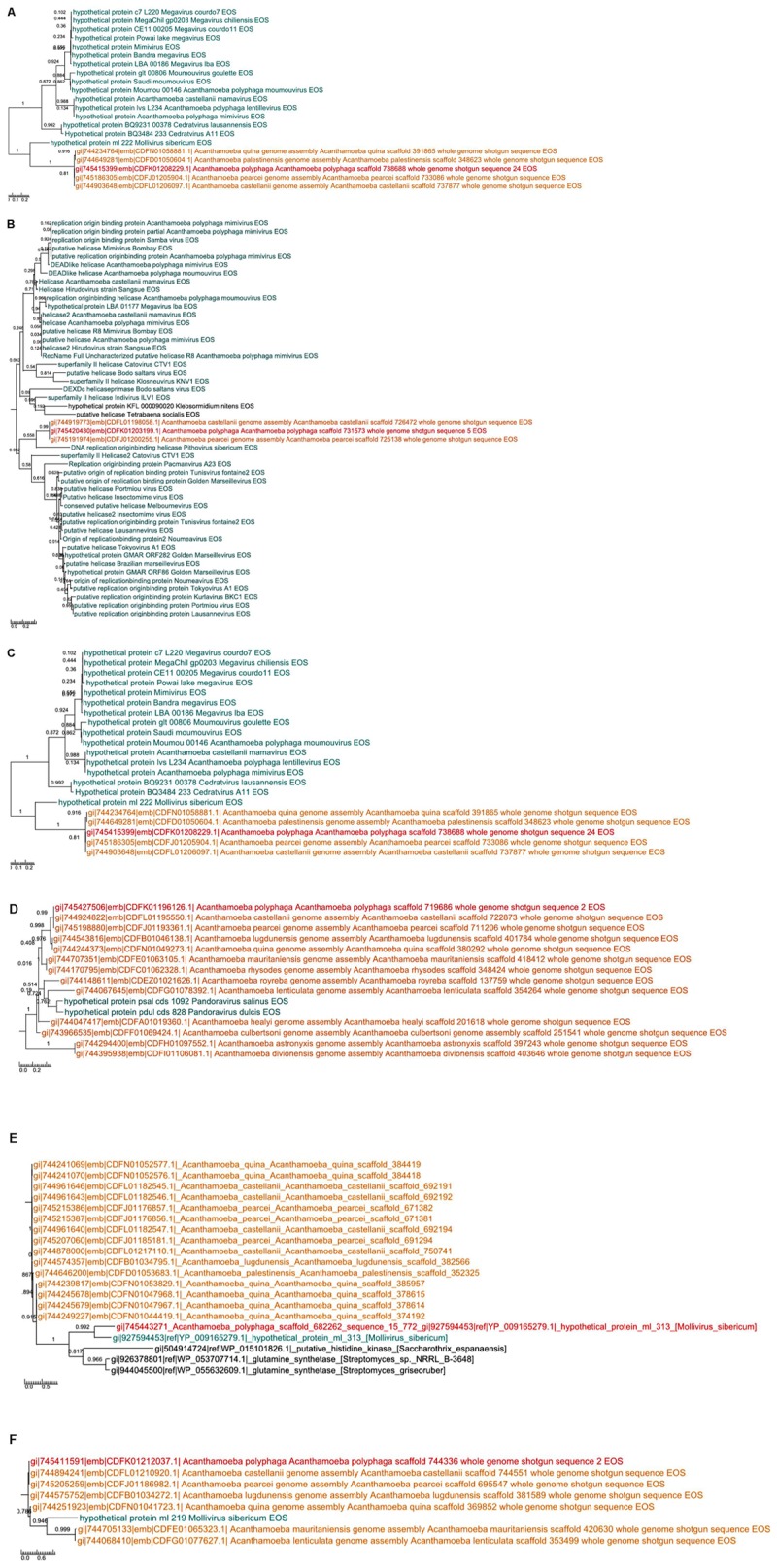
Phylogenetic trees for six examples of putative viral proteins in *A. polyphaga.* Gene sequence transfer was inferred from the comparison between annotated sequences with a putative viral origin and their best hits as well as their homologs in the other *Acanthamoeba* genomes. The way of the transfer was supposed to be from giant viruses to amoebae **(a–c)** or from amoebae to giant viruses **(d–f)**. Trees are unrooted. In red: *A. polyphaga* gene; in blue: the viral homolog; in orange homologs from other *Acanthamoeba* species; in black: homologs from other organisms.

**FIGURE 4:**
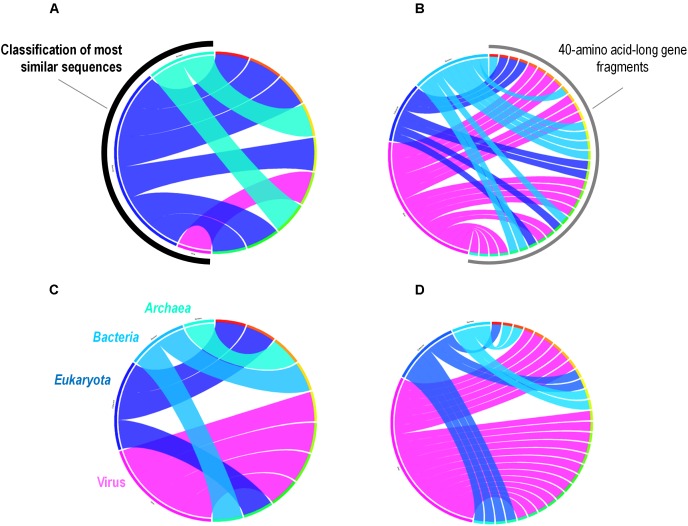
Rhizomes of *A. polyphaga* gene fragments illustrative of the mosaicism of the genes. Forty amino acid-long fragments of four *A. polyphaga* genes either supposed to have been transferred from viruses to amoebae **(A,B)** or in the opposite way **(C,D)** were linked to their most similar sequences in the NCBI GenBank protein sequence database according to the BLAST program (https://blast.ncbi.nlm.nih.gov/Blast.cgi), classified according to their belonging to viruses, eukaryotes, bacteria or archaea, and integrated in a circular gene data visualization. The figures were performed using the CIRCOS online tool (http://mkweb.bcgsc.ca/tableviewer/visualize/).

A total of 262 *A. polyphaga* ORFs had as best hit a coding sequence of a giant virus of amoeba, among which 134 are larger than 100 amino acids and 115 are present in scaffolds harboring a majority of ‘non-viral’ ORFs. For these 115 ORFs, results from BLAST searches against the non-redundant GenBank protein sequence database and all 14 *Acanthamoeba* draft genome sequences showed different patterns of best hits, including sequence sets comprising a majority of sequences from giant viruses or from *Acanthamoeba* spp. Subsequent phylogenetic reconstructions enabled us to infer that the most parsimonious evolutionary scenario was, in at least three cases, a gene sequence transfer from giant viruses of amoebae to *Acanthamoeba* spp., although alternative scenarios could not be ruled out (**Figures 3A–C**). Also, in at least three cases, gene sequence transfer was supposed to have occurred in the opposite way, from *Acanthamoeba* spp. to giant viruses of amoebae (**Figures 3D–F**). Nevertheless, phylogenies were most often inconclusive regarding the putative sense of the gene flow (**Supplementary Figure S5**). We analyzed further two cases for which the putative sense of the gene sequence transfer was from giant viruses to amoebae and two cases for which the putative sense of the gene sequence transfer was from amoebae to giant viruses. We searched for the most similar sequences for short fragments of these genes. We found that the best hits for these fragments were organisms that belonged to different cellular domains (*Eukarya*, *Bacteria*, or *Archaea*), or were of putative viral origin (**Figure 4**). This gene sequence mosaicism was observed in all four cases, although with level differences. This indicates that sequence mosaicism may also occur within genes, and may challenge the interpretation of gene-based phylogeny.

As previous culture isolation experiments suggested different levels of permissivity to giant viruses according to the *Acanthamoeba* species (Dornas et al., 2015), sequences detected in *A. polyphaga* and *A. castellanii* ATCC 50370 that were homologous with giant viral genes were compared (**Supplementary Table S6** and **Supplementary Figure S6**). This showed that the numbers and sets of viral genes that are homologs of genes present in these two *Acanthamoeba* species differ between giant viral families. In contrast, for a given viral family, a majority of genes found as best matches of genes from these two *Acanthamoeba* species were conserved in different viruses. Nonetheless, in some viruses, we identified genes that are homologous with only one of the two *Acanthamoeba* species. For example, homologs of some mimivirus genes were specifically found in *A. polyphaga* (**Supplementary Table S6**). Furthermore, analysis of the presence and conservation of these genes in the draft genome sequences of the other *Acanthamoeba* species showed that some were present in a majority of these genomes, being only absent in those of two or three other *Acanthamoeba* species. For instance, Pandoravirus salinus gene Ps_2278 was only absent in *A. castellanii* ATCC 50370 and *A. healyi*, whereas Pandoravirus dulcis gene Pd_13-16 was absent in *A. polyphaga*, *A. mauritaniensis*, and *A. pearcei*. In contrast, no giant virus homologous genes were found in the draft genome sequences of a substantial number of *Acanthamoeba* species, including pandoravirus genes Ps_1170 and Pd_589. In addition, the same gene in the draft genome sequences of *A. castellanii* ATCC 50370 was homologous with both Pandoravirus salinus gene Ps_2319 and Pandoravirus dulcis gene Pd_1426 (**Supplementary Table S7**).

*Acanthamoeba polyphaga* is one of the *Acanthamoeba* species for which no MCP homologs were detected. In contrast, eight MCP homologs were found in the draft genome sequences of other *Acanthamoeba* species including *A. lenticulata*, *A. lugdunensis*, *A. quina*, *A. healyi*, and *A. mauritaniensis* (**Supplementary Figure S7** and **Supplementary Table S8**), as previously described (Maumus and Blanc, 2016). In addition, *A. castellanii* Neff was previously found to harbor six genes encoding MCPs of giant viruses (Maumus and Blanc, 2016). A phylogenetic analysis showed that three of these sequences, including MCP homolog 1, MCP homolog 2, and iridovirus MCP homolog 2, were clustered. Moreover, the iridovirus homolog 1 sequence is clustered with a sequence from Mollivirus sibericum, the nearest neighbor of the pandoraviruses for which no gene encoding a capsid protein has been identified (Legendre et al., 2015) (**Supplementary Figure S8a**). A BLAST search was performed against the NCBI non-redundant protein sequence database. The query used was the ancestral sequence inferred for the MCP protein detected in the draft genome sequences of the different *Acanthamoeba* species and the MCP of Mollivirus sibericum. This search retrieved MCP-encoding sequences from phycodnaviruses, which are DNA viruses that infect algae and are classified with giant viruses of *Acanthamoeba* in the proposed order Megavirales (**Supplementary Figure 8b**). The G + C content was homogenous along the *Acanthamoeba* scaffolds carrying these virus-related genes.

### Synteny of *Acanthamoeba polyphaga* Genes With Viral Genes as Best Hits in the Draft Genome Sequences of the 16 *Acanthamoeba* spp.

**FIGURE 5:**
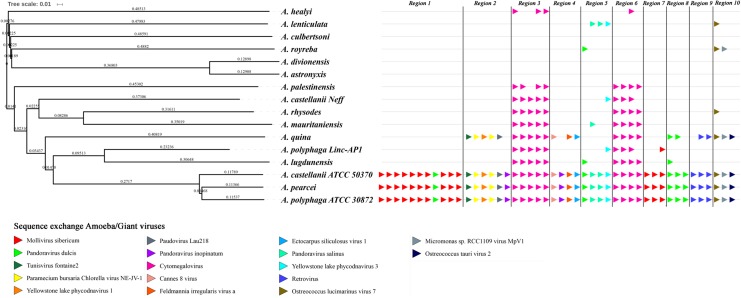
Comparison between gene synteny for genes with giant virus genes as best match in the genome sequence of 16 *Acanthamoeba* isolates classified in 14 species, and genome tree built for these *Acanthamoeba* species. Phylogenetic tree using the complete draft genome sequences of the 16 *Acanthamoeba* strains was represented aside the synteny distribution of genes best matching with giant virus genes in 10 selected genomic regions from *Acanthamoeba* spp. Phylogenetic reconstructions were performed using the alignment of 16 draft genome sequences of *Acanthamoeba* strains classified in 14 species including two *A. polyphaga* strains and two *A. castellanii* strains, by using the progressive Mauve program (Darling et al., 2010). Pointing triangles on the right part of the Figure correspond to viral genes; viruses are represented by different colors.

The evolution of the 10 genomic regions identified among the draft genome sequences of *Acanthamoeba* spp. and carrying the highest number of genes best matching with viruses (at least 3 genes) was analyzed. This showed that the three amoebal species *A. polyphaga* ATCC30872, *A. castellanii* ATCC 50370 and *A. pearcei* all conserved the 10 scaffolds that harbored in totality 46 viral genes. In addition, this analysis revealed a shared synteny with similar contents and co-localization of the viral-related genes in these three *Acanthamoeba* species. In contrast, for *A. quina, A. lugdunensis, A. mauritaniensis, A.* *rhysodes, A. palestinensis, A. healyi, A. lenticulata*, and *A. royreba*, the number of conserved genes was 24, 11, 10, 10, 8, 4, 4, and 3, respectively. In addition, none of these 46 genes was found in *A. culbertsoni*, *A. astronyxis* and *A. divionenesis* (**Figure 5**). The tree based on similarities between the 14 draft genome sequences and the genomes of *A. castellanii* Neff and *A. polyphaga* Linc-AP1 showed that the pattern of conservation of these genes homologous with viral genes in the draft genome sequences of the different *Acanthamoeba* species was congruent with the phylogenomic analyses since they displayed the same distribution into the groups of species.

## Discussion

We give here new insights into the possible interactions between *Acanthamoeba* species and giant viruses. Only one *A. castellanii* genome has been analyzed so far (Clarke et al., 2013). Our analyses primarily focused on *A. polyphaga*, for which the body of data regarding the isolation and propagation by culture of giant viruses is the greatest among amoebal species (along with that of *A. castellanii*). Great similarities were suggested between *A. polyphaga* and *A. castellanii* Neff, as *A. polyphaga* was found to harbor the majority of the 14,974 genes previously predicted through genome and transcriptome sequencing in *A. castellanii* Neff (Clarke et al., 2013). In contrast, it is worthy to note that *A. castellanii* Neff and *A. castellanii* ATCC 50370, whose draft genome sequences were analyzed here, did not cluster together based on our phylogenetic analyses. Furthermore, there is possibly an incorrect identification of sequences presented as originating from ATCC 30872 but belonging to *A. polyphaga* species. Indeed, the 18S ribosomal DNA previously described (AY026244) and part of the draft genome sequences analyzed here were not the closest among sequences from different *Acanthamoeba* species, and were not clustered in the phylogenetic analysis. These issues deserve clarification in future studies.

More than half of the genes predicted from the *A. polyphaga* draft genome sequences analyzed here had no homologs in the NCBI non-redundant sequence database. This suggests that a large part of the genome sequence of this amoeba remains unknown. Yet, a significant proportion of the predicted ORFs contained less than 100 codons. They should be considered carefully, especially when identified as ORFans or annotated as hypothetical proteins. Among annotated genes, the vast majority had homologs in amoebae, and 3% had homologs in other eukaryotes or in bacteria. Finally, a small proportion of annotated genes was mostly related to viral sequences, among which a majority belonged to the three pandoraviruses described to date and to *Mollivirus sibericum*.

The presence of sequences homologous with coding sequences of giant viruses of amoebae in eukaryotic genomes has been described by several teams (Filée, 2014; Maumus et al., 2014; Blanc et al., 2015; Sharma et al., 2016; Maumus and Blanc, 2016; Gallot-Lavallée and Blanc, 2017). Notably, MCPs were recently unexpectedly described in some *Acanthamoeba* species (Clarke et al., 2013; Maumus and Blanc, 2016; Gallot-Lavallée and Blanc, 2017). Congruently, our analysis showed that these sequences comprised two groups: firstly, the sequence of the “iridovirus-like” MCP of *A. castellanii* Neff and a sequence of Mollivirus sibericum, and, secondly, the other sequences which are related to those of a phycodnavirus (genus *Raphidovirus*), Heterosigma akashiwo virus 01. The G+C content was found to be homogeneous along the *Acanthamoeba* scaffolds carrying these genes best matching MCPs. However, this does not completely rule out the possibility that these sequences are the result of an exchange of sequences between these organisms because the GC% in some giant viruses is relatively close to that of their host, for example for pandoraviruses and *A. castellanii* (60.6% vs. 58.3%) (Antwerpen et al., 2015). These results also confirm previous observations concerning the presence of homologous sequences of MCPs in the genome of some eukaryotes (Maumus et al., 2014). Thus, these data suggest nucleotide sequence transfers between giant viruses and *Acanthamoeba*, and raise the question of the significance of homologies between genes present in giant virus and *Acanthamoeba* genomes. One hypothesis is that capsid proteins may be involved in defense mechanisms. Interestingly, it has been described that, in addition to their function as capsid proteins, the MCPs of totivirus, a RNA virus, was also able to inactivate the host mRNAs by eliminating their 5’-cap (Koonin, 2010).

Preliminary analysis of possible nucleotide sequence transfers within the *A. polyphaga* genome showed that there are 366 genes that could have been exchanged between this amoeba and viruses. We compared this gene set with the set of 267 genes recently described in *A. castellanii* Neff as putatively exchanged with viruses (Maumus and Blanc, 2016). We found that among these 366 *A. polyphaga* genes, only 30 (8.2%) were shared with the 267 genes identified in *A. castellanii* Neff as putatively exchanged with viruses. In addition, only 39 (11.0%) among the 356 genes which had as best hit a coding sequence of viruses in the draft genome sequences of *A. castellanii* ATCC 50370 were shared with the 267 genes described by Maumus and Blanc (Maumus and Blanc, 2016). A total of 11% of the 366 genes were matching with genes transcribed in *A. castellanii* Neff (Clarke et al., 2013), suggesting that they might be transcribed and play yet unknown roles. Some of these differences might be explained by differences between the sets of giant virus genomes available at the time when the different analyses were performed, as giant virus diversity expands considerably (Maumus and Blanc, 2016; Colson et al., 2017b). In addition, differences in parameters used for gene prediction and annotation and BLAST searches are likely to generate discrepant results. It should be also taken into account that the amoebal genomes analyzed here were non-assembled draft genome sequences. Among giant viruses which infect *Acanthamoeba* spp., pandoraviruses were found to share the highest number of genes with *A. polyphaga*, before mimiviruses and marseilleviruses, with 117, 65 and 24 putatively exchanged genes, respectively. This might suggests a co-evolution of *A. polyphaga* with pandoraviruses, but it is worth considering that this *Acanthamoeba* species was far less permissive to pandoraviruses than *A. castellanii* (Dornas et al., 2015). In addition, there is little evidence of gene exchanges with other viruses, including phycodnaviruses. The sense of the gene nucleotide sequence transfers remained undetermined in a large majority of cases. This is due to an insufficient number of matches, or an absence of significantly delineated cluster in the phylogenetic trees. Nevertheless, for some of the phylogenies, a transfer from *Acanthamoeba* to giant viruses was the most parsimonious evolutionary hypothesis, indicating that giant viruses are not only bags of genes but contribute to the gene sequence flow. Estimating times of divergence might be helpful in more extensive analyses that would be conducted on giant viruses of amoebae and several *Acanthamoeba* species in order to try inferring the sense of gene sequence transfers between giant viruses and their amoebal hosts. Besides, the analyses of fragments of *A. polyphaga* genes that had a giant virus sequence as best hit showed a different, more complex pattern of best hits compared to the analysis of the whole genes, with mosaics of sequences from eukaryotes, bacteria, archaea and giant viruses as best matches. A similar pattern has also been illustrated by fragmenting a gene encoding an aminoacyl-tRNA synthetase in Klosneuvirus, a mimivirus relative (Abrahao et al., 2018). These findings may extend to genes the paradigm that no single tree can define the mosaic origin of genomes, which may result from nucleotide sequence transfers rather than from gene transfers (Dagan and Martin, 2006; Merhej et al., 2011). These mosaic patterns that affect the sequences of genes may represent a pitfall for the robustness of phylogenetic analyses and inference of the putative way of nucleotide sequence transfer. This further suggests that the vision of species in Darwin’s tree of life is rather outdated, and that each organism has a complex family tree that is a testimony of its chimerical origin and is better represented in the form of a rhizome than of a tree (Raoult, 2010; Merhej et al., 2011). Overall, the global analysis of all giant virus homologs to *A. polyphaga* predicted genes potentially demonstrates the complexity of the putative gene trafficking between this amoeba and giant viruses, with 1,797 genes involved, although only 366 *A. polyphaga* genes have viral genes as best matches. This might further suggest intermediate interactions with organisms other than viruses. Moreover, these results highlight the fact that the gene flow was not a one way mechanism and likely resulted from the sympatric lifestyle of giant viruses in amoebae (Moliner et al., 2010).

The comparisons of possible gene sequence transfers between the two species *A. polyphaga* and *A. castellanii* ATCC 50370 and some representatives of giant viruses (pandoraviruses, *Acanthamoeba polyphaga* mimivirus and Marseillevirus) shows that the differences regarding the number and the nature of potentially transferred genes remain limited within the same viral family. However, the number and the nature of potentially transferred genes were found to be more variable when considering different families of giant viruses. These observations might be explained by differences in the frequency of interactions between some *Acanthamoeba* species and some giant viruses, and between genes used by both the amoebae and the giant viruses to deal with these interactions. The recent study by Clarke et al. (2013) showed that the plasticity of protists living in community with microbes is as important as that of bacteria with the same lifestyle. This supports the hypothesis that an essential explanation of the chimerism of the genomes of organisms and micro-organisms that live sympatrically relies on this lifestyle, and possibly on their genomic plasticity according to their phylogenetic origin (eukaryotic, bacterial, archaeal or viral). These observations may also reflect the time of sequence exchange through the evolutionary course of both giant viruses and amoeba species. Phylogenetic and synteny analyses of viral genes performed in the present study suggest that the sequence exchanges between *Acanthamoeba* species and giant viruses occurred along the *Acanthamoeba* speciation events and evolved through species-specific events. Among the *Acanthamoeba* species, *A. castellanii* ATCC 50370 and *A. pearcei* were found to be the most structurally conserved with *A. polyphaga* regarding their content of genes homologous with viral genes. These results did follow the expectation that decreased phylogenetic distance would correspond to increased levels of genome preservation.

In summary, this work opens up several potential avenues for future works on the interactions between *Acanthamoeba* species and giant viruses. The annotation of all *Acanthamoeba* species and their accurate identification are important tasks for a greater understanding of why some amoebae are more susceptible than others to giant viruses, and possibly to other microorganisms. Thus, the considerable diversity of gene repertoires among *Acanthamoeba* species might lead to differences regarding potential interactions with giant viruses. The characterization of the genes present and absent in the different species of *Acanthamoeba* could be performed and correlated with the observed phenotypic differences that need to be studied more extensively.

## Author Contributions

PC and BLS designed the study. NC, PC, and AL performed the experiments. NC, PC, AL, PP, DR, and BLS analyzed the data. All the authors contributed to manuscript redaction and review.

## Conflict of Interest Statement

The authors declare that the research was conducted in the absence of any commercial or financial relationships that could be construed as a potential conflict of interest.

## Footnotes

1 http://www.ncbi.nlm.nih.gov/bioproject/

2 https://www.qiagenbioinformatics.com/products/clc-genomics-workbench/

3 http://www.megasoftware.net/

4 http://circos.ca/
