# Supplementary figures and images for: A Phylogenomic Study of Acanthamoeba polyphaga Draft Genome Sequences Suggests Genetic Exchanges With Giant Viruses
Source: Front Microbiol. 2018 Sep 6;9:2098. doi: 10.3389/fmicb.2018.02098 (PMC6135880; doi:10.3389/fmicb.2018.02098)

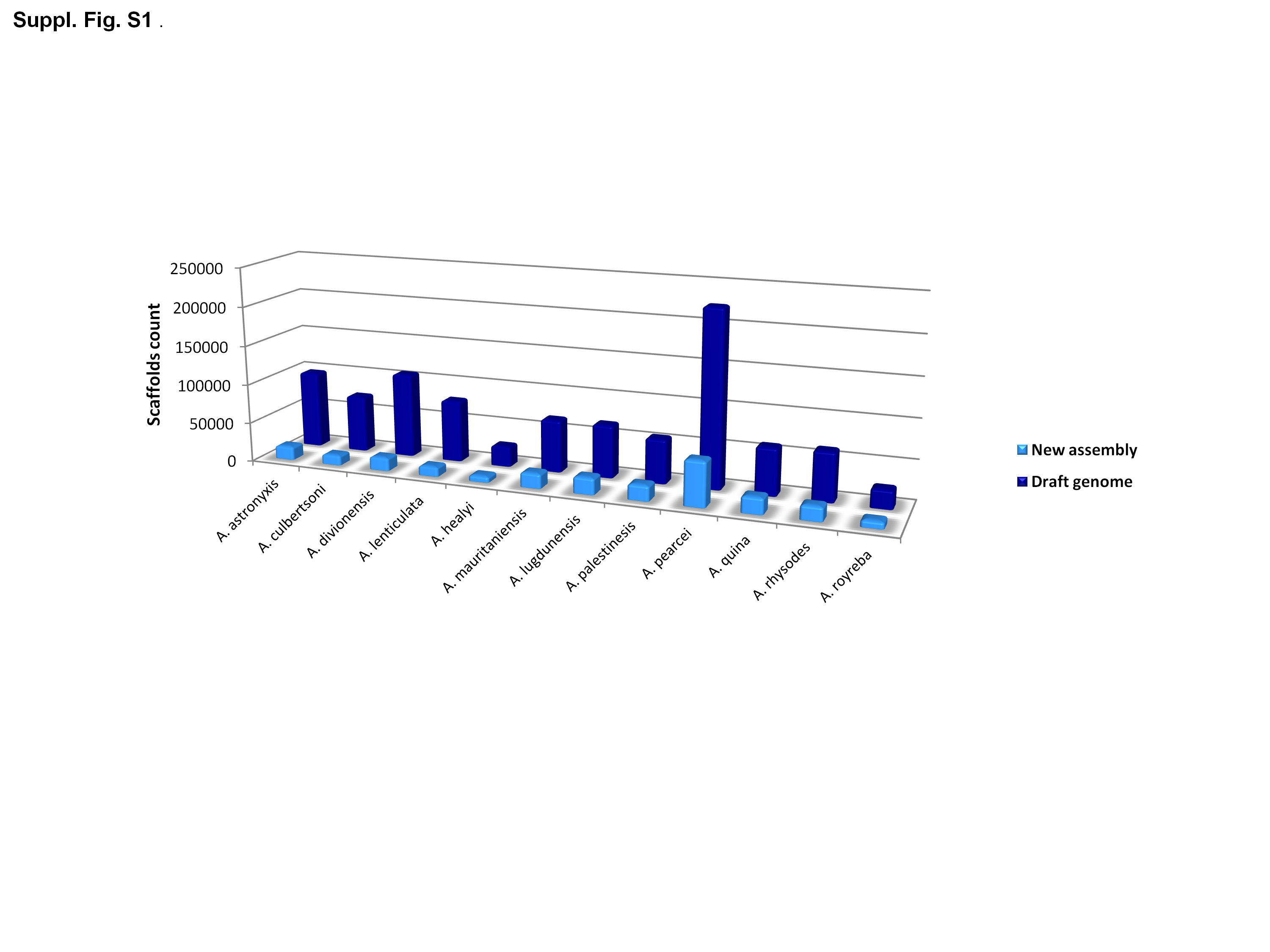

Supplement: Supplementary file 1 [file Image_1.TIF]

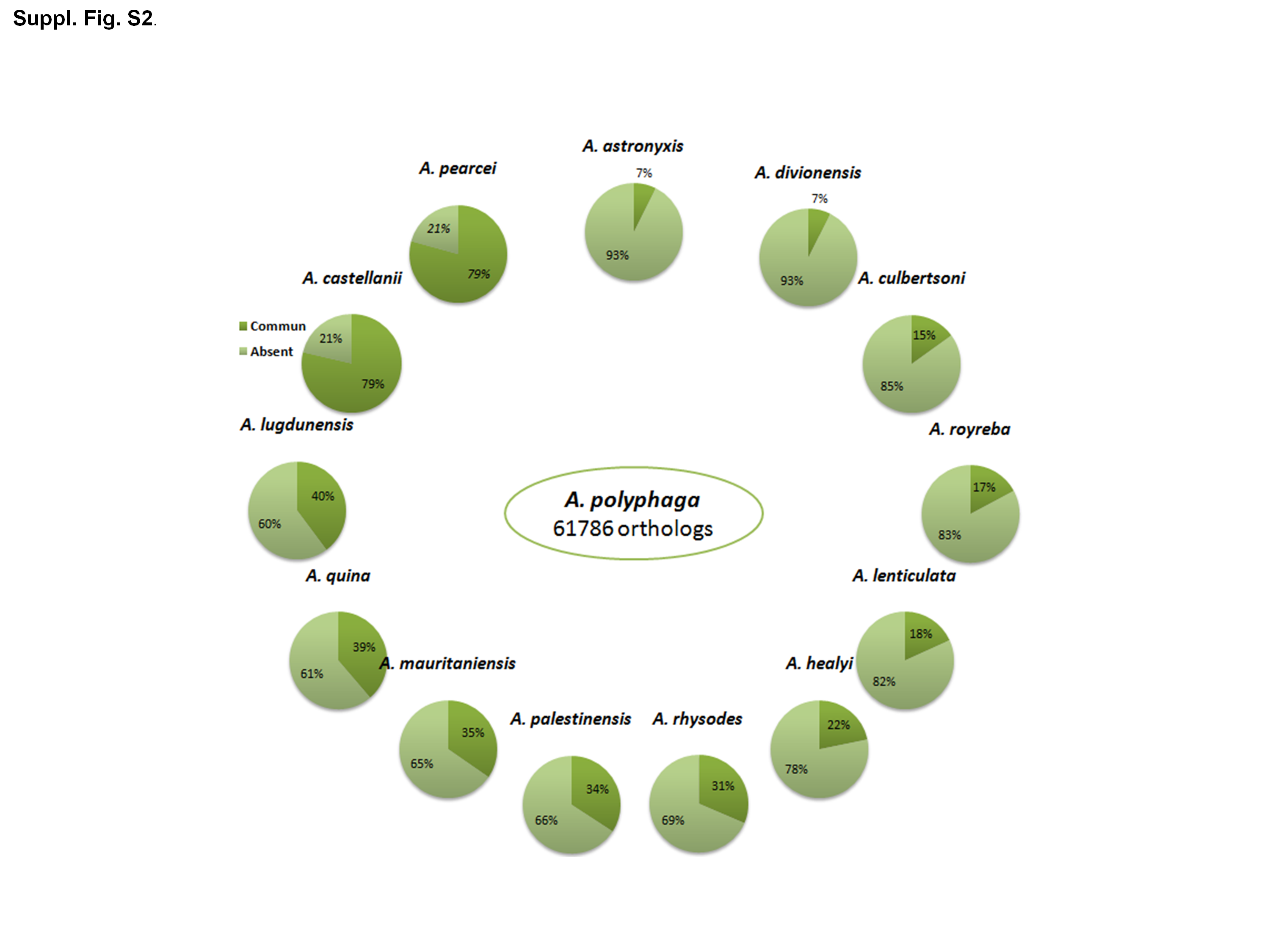

Supplement: Supplementary file 2 [file Image_2.TIF]

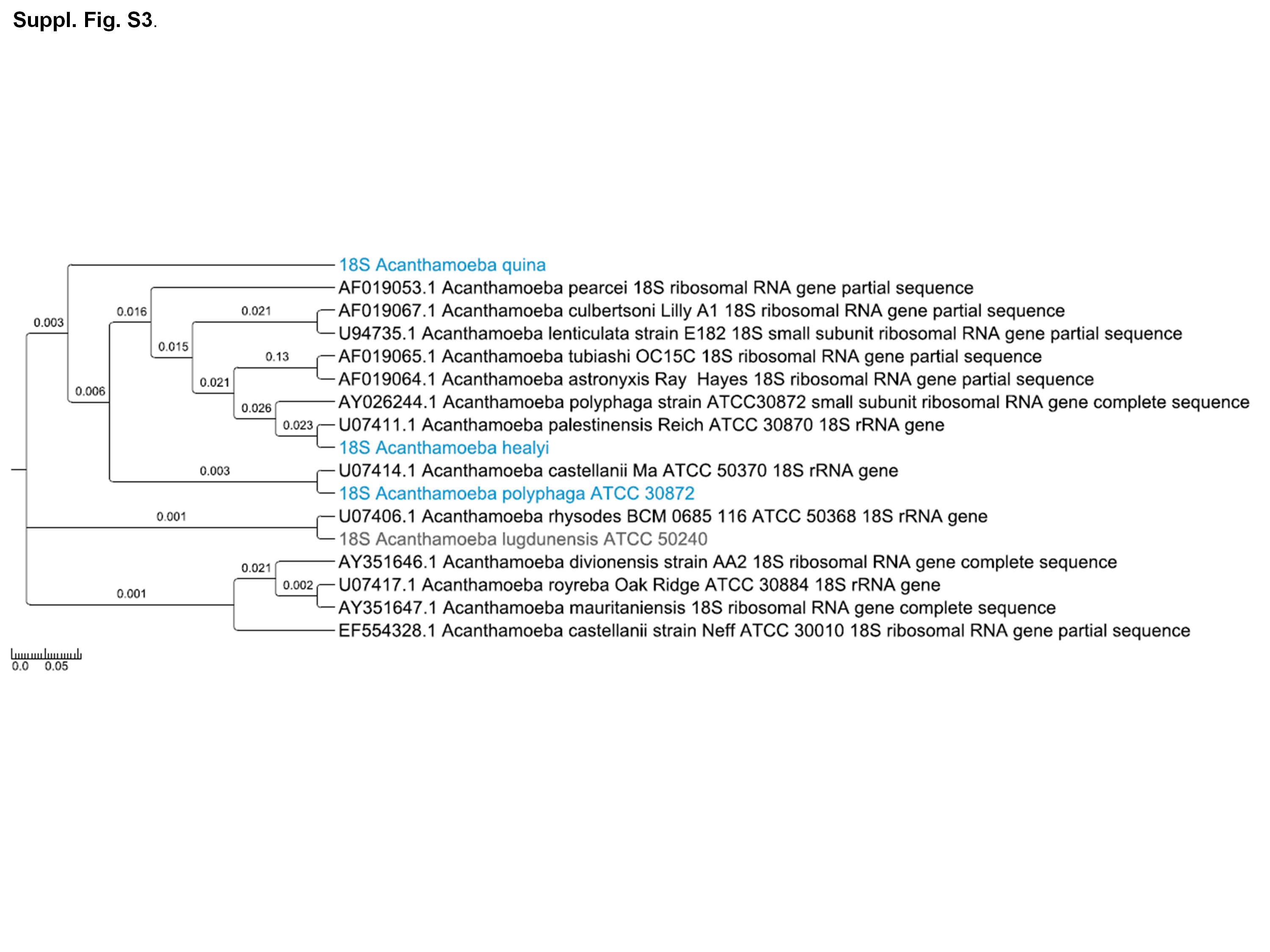

Supplement: Supplementary file 3 [file Image_3.TIF]

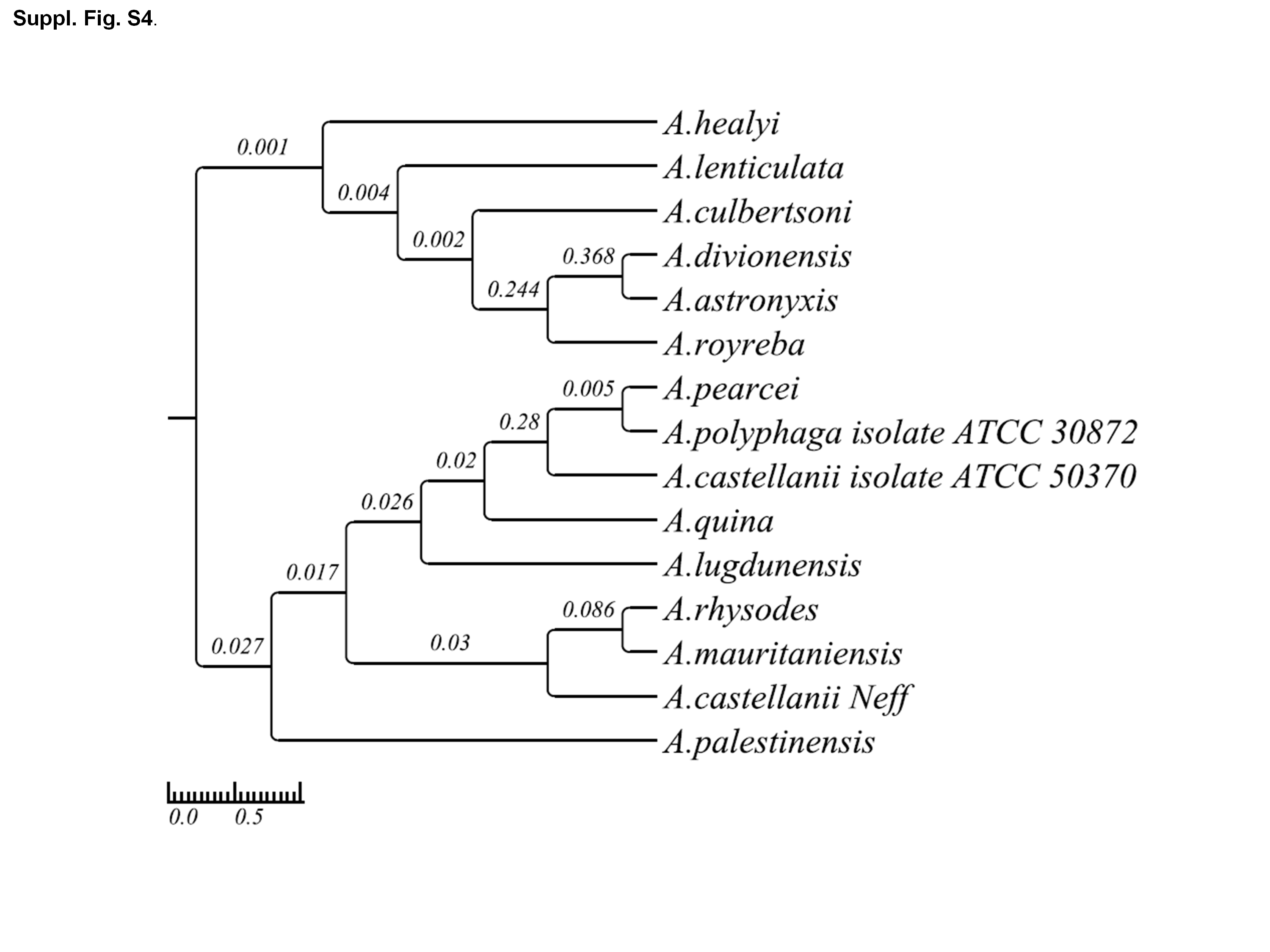

Supplement: Supplementary file 4 [file Image_4.TIF]

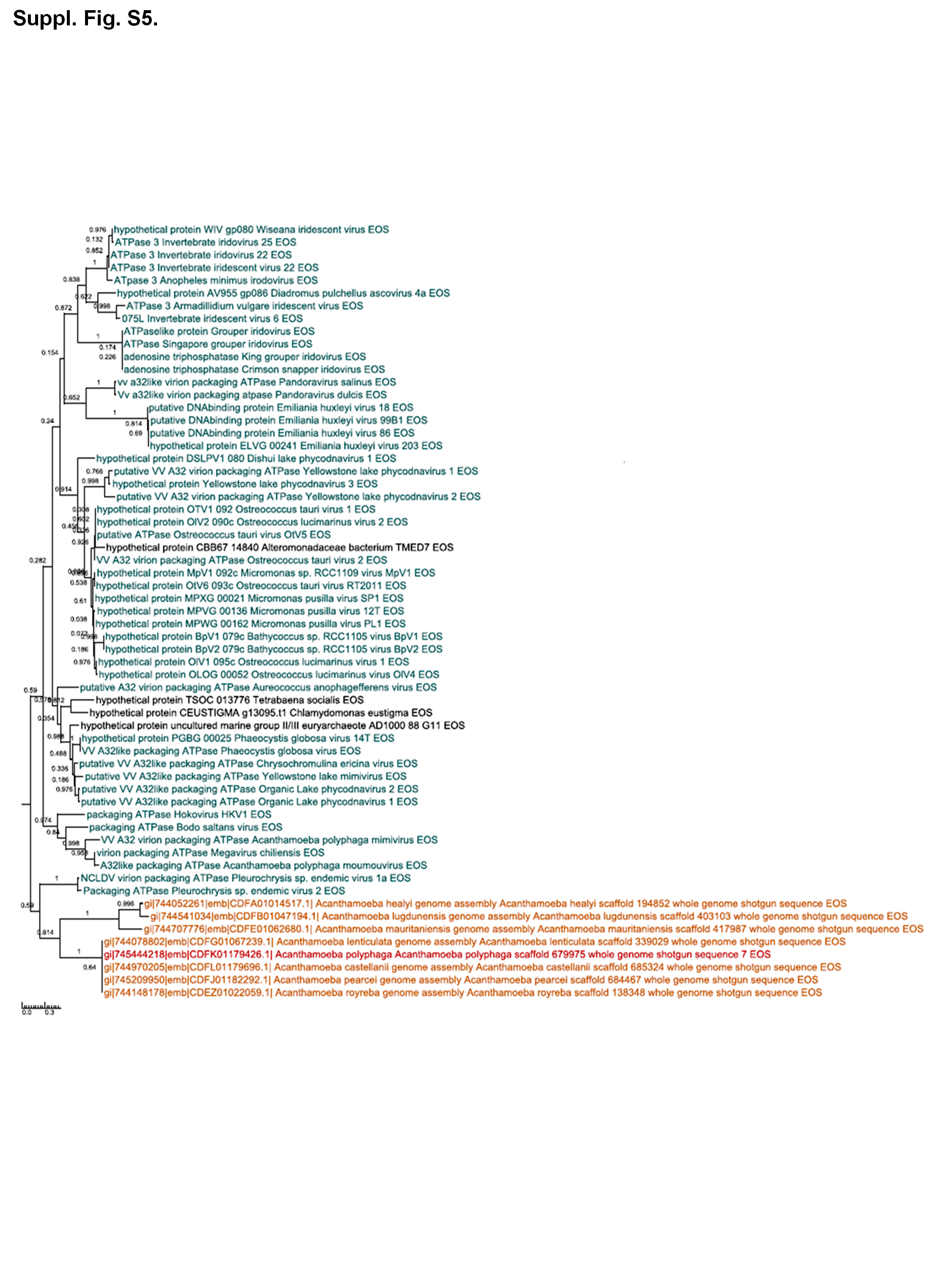

Supplement: Supplementary file 5 [file Image_5.TIF]

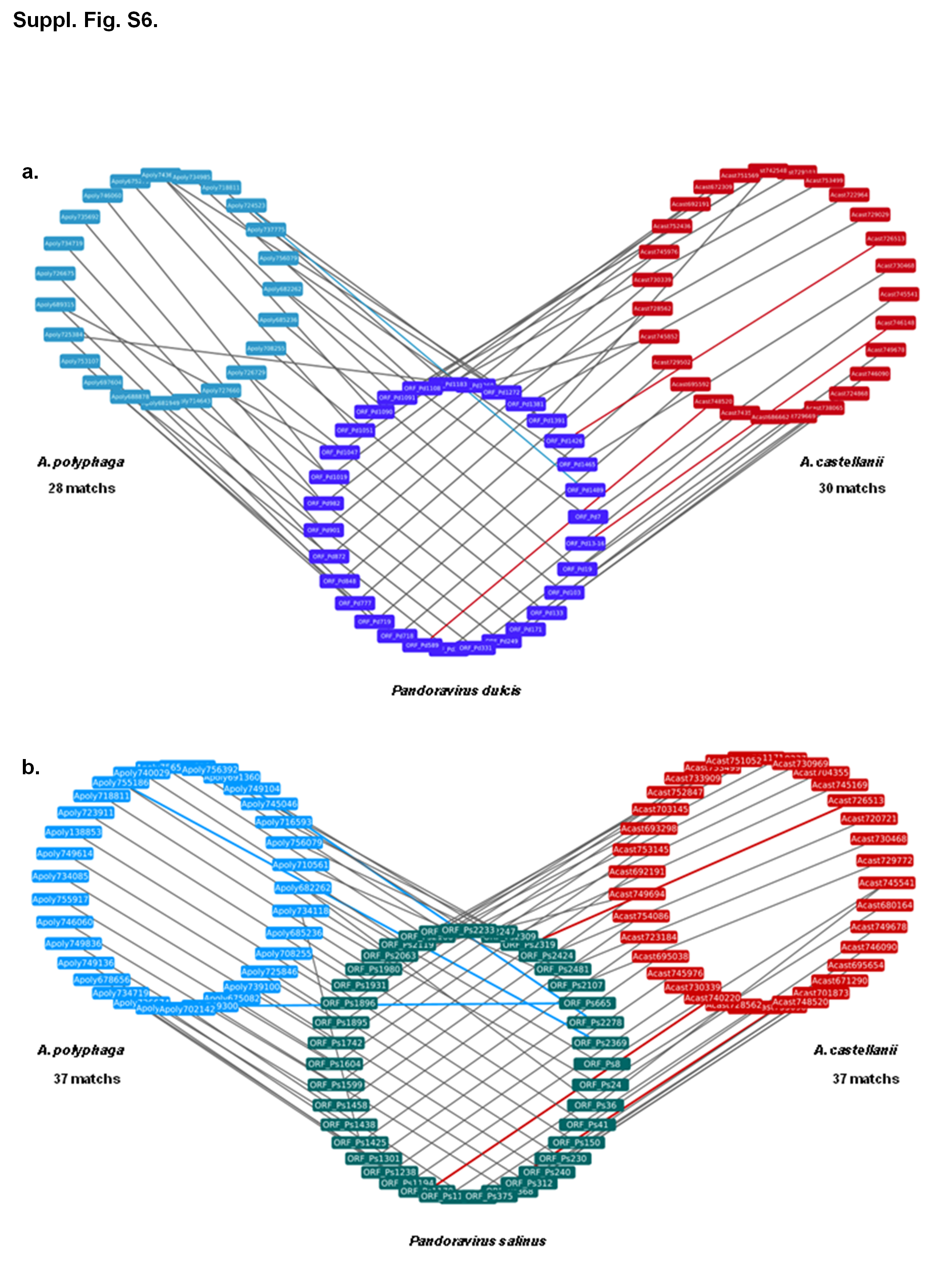

Supplement: Supplementary file 6 [file Image_6.TIF]

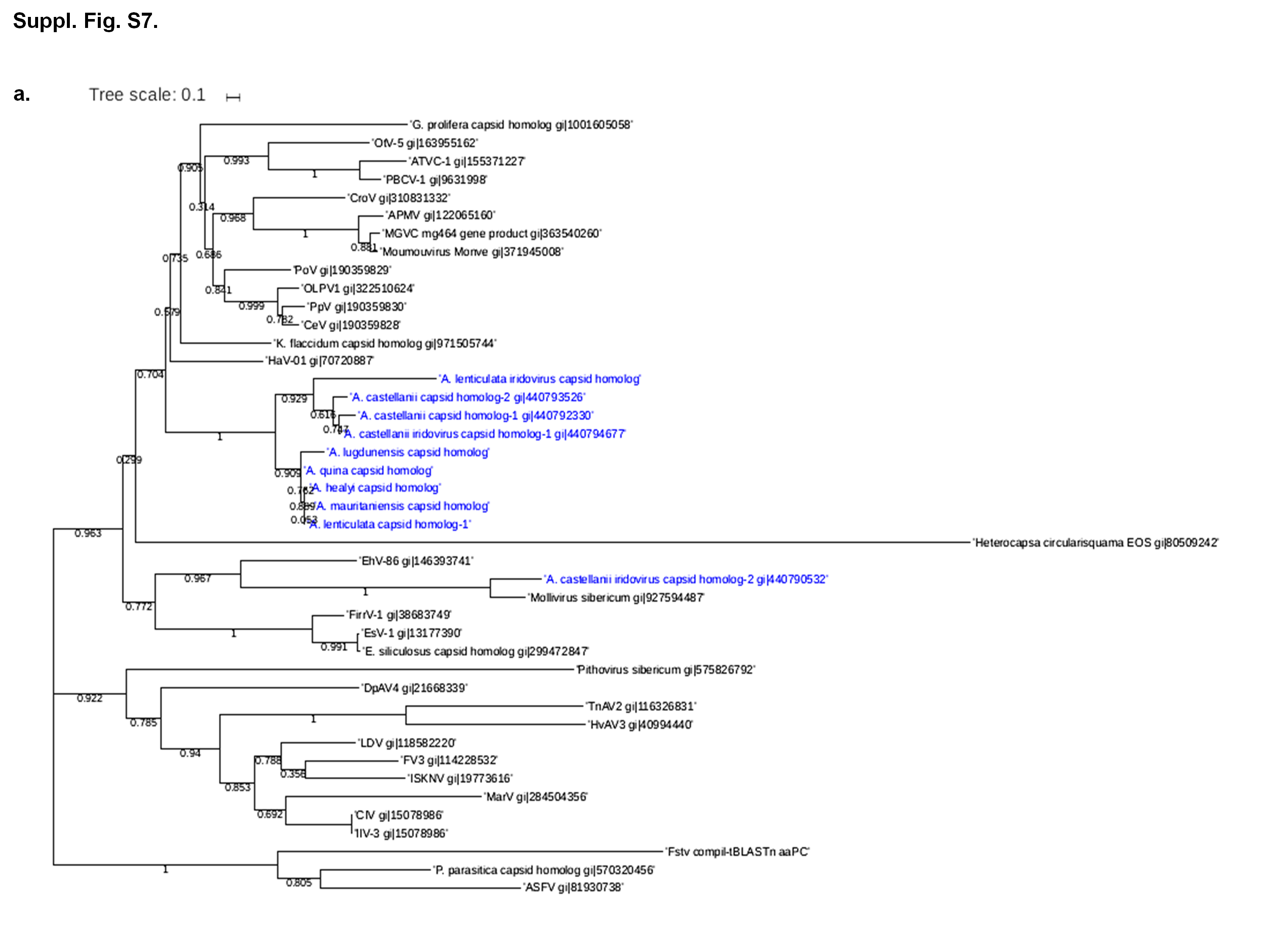

Supplement: Supplementary file 7 [file Image_7.TIF]

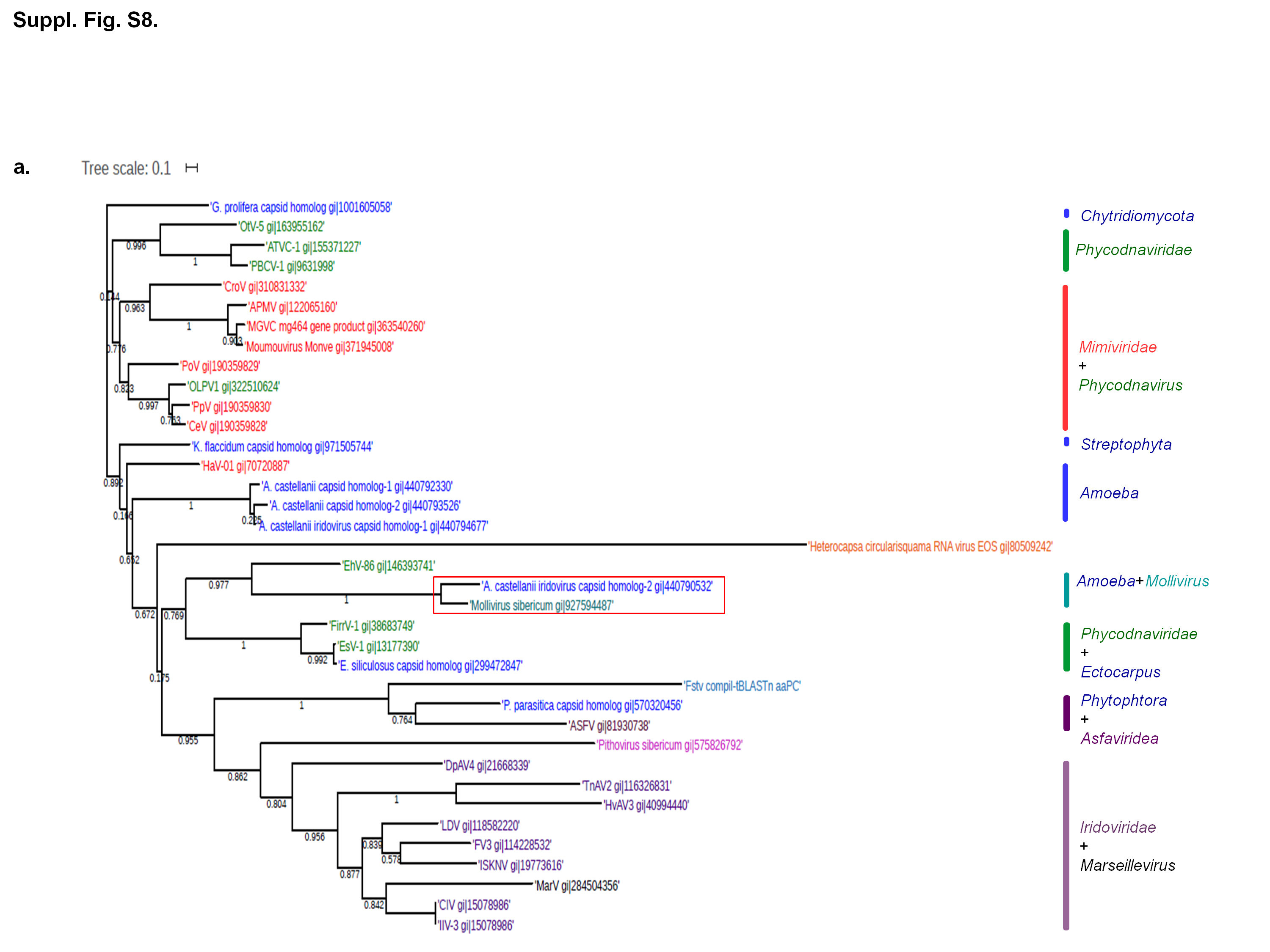

Supplement: Supplementary file 8 [file Image_8.TIF]

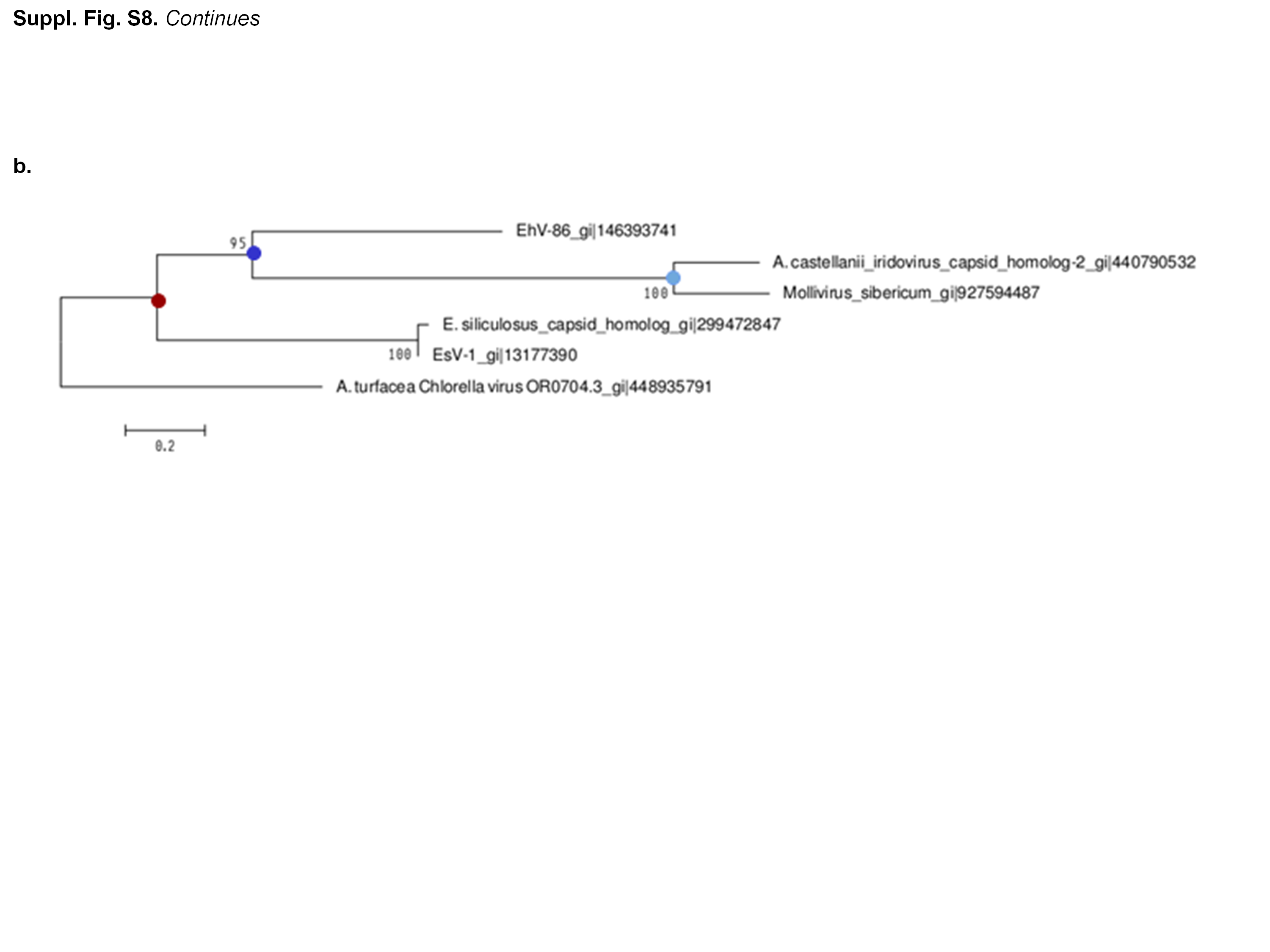

Supplement: Supplementary file 9 [file Image_9.TIF]
