# Supplementary material for: A Phylogenomic Study of Acanthamoeba polyphaga Draft Genome Sequences Suggests Genetic Exchanges With Giant Viruses
Source: Front Microbiol. 2018 Sep 6;9:2098. doi: 10.3389/fmicb.2018.02098 (PMC6135880; doi:10.3389/fmicb.2018.02098)
Supplement: Supplementary file 10 [file Table_1.docx]

**SUPPLEMENTARY MATERIAL**

**Supplementary Figures**

**Fig. S1.** Comparison of the number of scaffolds before (dark blue) and after assembly (light blue) using the CLC software for 12 other species of *Acanthamoeba.*

**Fig. S2.** Analysis of the gene repertoires for the 14 draft genomes sequences of *Acanthamoeba*, and identification of the gene groups shared with *A. polyphaga* and those which were absent in the draft genome sequences of this species.

**Fig. S3.** Phylogenetic reconstruction based on 18S ribosomal genes. 18S DNA sequences for different *Acanthamoeba* strains were obtained by sequencing the complete 18S (dark grey) from NCBI (black) or directly from the draft genomes sequences (blue) for non available strains.

Phylogenetic reconstructions were performed with the 18S ribosomal DNA sequences from 33 different *Acanthamoeba* strains, including those of the 16 *Acanthamoeba* strains classified in 14 *Acanthamoeba* species. The 18S ribosomal DNA sequences were obtained by sequencing the complete 18S ribosomal DNA from strains available in our laboratory, and for non-available strains, the sequences were retrieved from the NCBI GenBank database or directly from the *Acanthamoeba* draft genome sequences.

**Fig. S4.** Tree based on the 14 draft genomes sequences of *Acanthamoeba* species.

Draft genomes sequences were aligned between each other before tree construction by the Mauve software (http://darlinglab.org/mauve/download.html)

**Fig. S5.** Phylogenetic trees for an example of putative viral protein in *A. polyphaga*.

Gene sequence transfer was inferred from the comparison of annotated sequences from putative viral origin with their best hits as well as their homologs in the other *Acanthamoeba* genomes.

The phylogeny was inconclusive to infer a sense for the transfer of gene sequence.

In red: *A. polyphaga* gene; in blue: the viral homolog; in orange homologs from other *Acanthamoeba* species; in black: homologs from other organisms.

**Fig. S6.** Network of *A. polyphaga* (in blue) and *A. castellanii* isolate ATCC 50370 (red) with Pandoravirus dulcis (a) and Pandoravirus salinus (b).

The number of viral genes for which a homolog is detected in the draft genomes sequences studied is indicated. The red edges correspond to the viral genes specific to *A. castellanii* and the blue edges correspond to the specific viral genes of *A. polyphaga*.

**Fig. S7.** Phylogenetic tree of the MCPs by adding the six homologs of MCPs in the draft genomes sequences of *Acanthamoeba* (in blue next to the four homologous sequences of the MCPs previously published in the Clarke et al. 2013).

**Fig. S8.** Phylogenetic trees for capsid proteins (MCPs) in *A. castellanii* Neff.

a: homologous sequences of the MCPs in *A. castellanii* str. Neff(dark blue) and MCP sequences of some giant virus representatives (highlighted according to their affiliation to viral families); as well as homologs of MCPs in other eukaryotes (dark blue). b: Focus on the group of sequences in the red box in Figure 8a, and their closest neighbors. The ancestral sequences (in dark blue, light blue and red) were predicted and included in the phylogenetic analysis.

**Supplementary Tables**

**Table S1.** Main representatives of giant viruses.

| Family/Taxon | Sublineage | Name | Genome Accession no. | Genome size (kbp) | Gene number | GC% |
| --- | --- | --- | --- | --- | --- | --- |
| *Mimiviridae* | A | Acanthamoeba polyphaga mimivirus | NC_014649.1 | 1,182 | 979 | 28.0 |
|  | A | Acanthamoeba castellanii mamavirus | JF801956.1 | 1,191 | 1059 | 28.0 |
|  | B | Acanthamoeba polyphaga moumouvirus | NC_020104.1 | 1,021 | 930 | 24.6 |
|  | C | Megavirus chiliensis | NC_016072.1 | 1,259 | 1,123 | 25.2 |
|  | C | LBA111 virus | JX885207.1 | 1,230 | 1,183 | 25.3 |
|  | Outgroup | Cafeteria roenbergensis virus | NC_014637.1 | 617 | 544 | 23.3 |
| *Marseilleviridae* | A | Marseillevirus marseillevirus T19 | NC_013756.1 | 368 | 457 | 44.7 |
|  | A | Senegalvirus | JF909596.1-602.1 | 373 | 479 | - |
|  | B | Lausannevirus | NC_015326.1 | 347 | 444 | 42.9 |
|  | C | Tunisvirus | KF483846.1 | 380 | 484 | 43.0 |
|  | C | Insectomime virus | KF527888.1 | 387 | 477 | 42.7 |
|  | D | Brazilian marseillevirus | NC_029692.1 | 362 | 491 | 43.3 |
| Pandoravirus |  | Pandoravirus salinus | NC_022098.1 | 2,474 | 2,544 | 61.7 |
|  |  | Pandoravirus dulcis | NC_021858.1 | 1,909 | 1,488 | 63.7 |
|  |  | Pandoravirus inopinatum | NC_026440.1 | 2,243 | 1,902 | 60.7 |
|  |  | Pandoravirus massiliensis | - | 1,595 | 1,415 | 60.1 |
| Pithovirus |  | Pithovirus sibericum | NC_023423.1 | 610 | 467 | 35.8 |
| Mollivirus |  | Mollivirus sibericum | NC_027867.1 | 651 | 523 | 60.1 |
| *Phycodnaviridae* |  | Organic Lake phycodnavirus 2 | HQ704803.1 | 466 | 457 | 36.2 |
|  |  | Phaeocystis globosa virus 12T | HQ634147.1 | 460 | 439 | 32.0 |
|  |  | Acanthocystis turfacea Chlorella virus 1 | NC_008724.1 | 456 | 495 | 37.8 |
|  |  | Ostreococcus virus OtV5 | NC_010191.1 | 186 | 247 | 44.8 |
|  |  | Paramecium bursaria Chlorella virus 1 | NC_000852.5 | 471 | 518 | 36.7 |
| *Iridoviridae* |  | Singapore grouper iridovirus | NC_006549.1 | 140 | 162 | 48.6 |
|  |  | Aedes taeniorhynchus iridescent virus | NC_008187.1 | 191 | 126 | 47.9 |
| *Poxviridae* |  | Cowpox virus | NC_003663.2 | 224 | 233 | 33.4 |
|  |  | Amsacta moorei entomopoxvirus L | NC_002520.1 | 232 | 294 | 17.8 |

**Table S2.** Results of gene prediction for the genomes of the 14 different species of *Acanthamoeba*.

| Species | Scaffolds sequence (Mb) | Total number of predicted ORFs | ORFs⩾ 35 aa | ORFs⩾ 50 aa | ORFs⩾ 100 aa |
| --- | --- | --- | --- | --- | --- |
| *Acanthamoeba castellanii* | 115.3 | 381,505 | 318,451 | 223,576 | 96,232 |
| *Acanthamoeba polyphaga* | 120.6 | 374,196 | 310,496 | 223,728 | 97,092 |
| *Acanthamoeba astronyxis* | 83.5 | 203,336 | 191,002 | 160,845 | 60,691 |
| *Acanthamoeba culberstoni* | 55.6 | 129,164 | 171,163 | 97,169 | 46,309 |
| *Acanthamoeba divionensis* | 84.6 | 211,955 | 198,459 | 166,060 | 61,225 |
| *Acanthamoeba healyi* | 75.3 | 161,883 | 154,995 | 136,395 | 67,989 |
| *Acanthamoeba lenticulata* | 66.1 | 153,534 | 142,638 | 118,847 | 58,461 |
| *Acanthamoeba lugdunensis* | 99.5 | 229,323 | 211,414 | 181,388 | 91,448 |
| *Acanthamoeba mauritaniensis* | 106.9 | 234,464 | 215,999 | 187,285 | 95,443 |
| *Acanthamoeba palestinensis* | 103.5 | 203,374 | 189,552 | 167,016 | 90,317 |
| *Acanthamoeba pearcei* | 115.8 | 386,877 | 321,604 | 228,394 | 95,162 |
| *Acanthamoeba quina* | 83.6 | 195,999 | 181,453 | 152,351 | 75,556 |
| *Acanthamoeba rhysodes* | 75.9 | 186,569 | 170,797 | 143,414 | 68,073 |
| *Acanthamoeba royreba* | 79.6 | 171,918 | 167,297 | 150,542 | 69,549 |

aa, amino acids

**Table S3.** Conserved proteins of *Acanthamoeba castellanii* Neff in *A. castellanii* isolate ATCC 50370 and *A. polyphaga.*

**Table S4.** Non-conserved proteins of *Acanthamoeba castellanii* Neff in *A. castellanii* isolate ATCC 50370 and *A. polyphaga*.

**Table S5.** List of the 366 and 356 genes with viral genes as best match in *A. polyphaga* and *A. castellanii* isolate ATCC 50370.

The number and nature of genes common with the 267 transferred genes in *A. castellanii* Neff, and the phylogenetic inference of *A. polyphaga* genes matching with viruses.

**Table S6.** Sequence exchanges between the two species of *Acanthamoeba*, *A. castellanii* isolate ATCC 50370 and *A. polyphaga*, and some representatives of giant viruses.

**Table S7.** Evaluation of the conservation for the genes which are specific to one of the two species, *A. castellanii* isolate ATCC 50370 and *A. polyphaga*, among those shared with Pandoravirus dulcis and Pandoravirus salinus in the other *Acanthamoeba* genomes.

The genes of P. dulcis are indicated in light blue and those of P. salinus are indicated in dark blue.

**
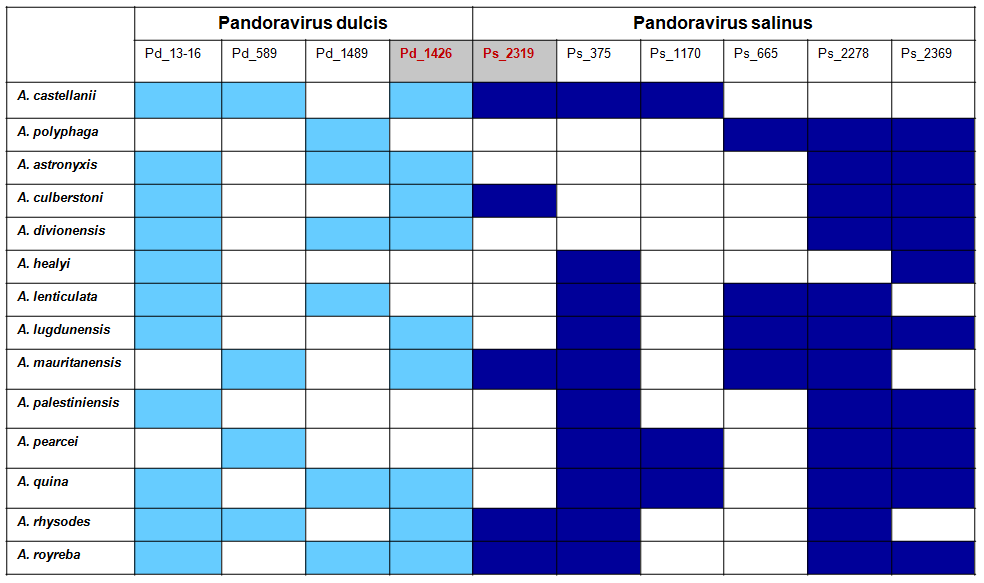
**

**Table S8.** Description of the hits for the eight *Acanthamoeba* sequences homologous to major capsid proteins (MCPs)

|  | *Acanthamoeba* species | Number of scaffolds | Amino acid identity (%) | E-value | Length (amino acids) |
| --- | --- | --- | --- | --- | --- |
| MCP homolog 3 | *A. healyi* | 205,704 | 97.3 | 0.0 | 255 |
|  | *A. lenticulata* | 353,499 | 95.3 | 4,00E-166 | 256 |
|  | *A. lugdunensis* | 402,197 | 86.3 | 7,00E-134 | 233 |
|  | *A. mauritaniensis* | 407,754 | 95.3 | 7,00E-165 | 256 |
|  | *A. quina* | 385,517 | 93.3 | 4,00E-135 | 180 |
| MCP homolog 2 | *A. lenticulata* | 353,569 | 69.1 | 1,00E-67 | 152 |
| MCP homolog Irido-like 1 | *A. lenticulata* | 343,630 | 67.8 | 2,00E-122 | 295 |
|  | *A. rhysodes* | 323,471 | 52.2 | 2,00E-42 | 182 |

MCP, major capsid protein
